# Control of directionality of chromatin folding for the inter- and intra-domain contacts at the *Tfap2c*–*Bmp7* locus

**DOI:** 10.1186/s13072-018-0221-1

**Published:** 2018-09-14

**Authors:** Taro Tsujimura, Osamu Takase, Masahiro Yoshikawa, Etsuko Sano, Matsuhiko Hayashi, Tsuyoshi Takato, Atsushi Toyoda, Hideyuki Okano, Keiichi Hishikawa

**Affiliations:** 1 Department of iPS Cell Research and Epigenetic Medicine, Keio University School of Medicine, 35 Shinanomachi, Shinjuku-ku, Tokyo 160-8582 Japan; 2 Department of Physiology, Keio University School of Medicine, 35 Shinanomachi, Shinjuku-ku, Tokyo 160-8582 Japan; 3 Apheresis and Dialysis Center, Keio University School of Medicine, 35 Shinanomachi, Shinjuku-ku, Tokyo 160-8582 Japan; 4 Department of Tissue Engineering, The University of Tokyo Hospital, 7-3-1 Hongo, Bunkyo-ku, Tokyo 113-8655 Japan; 5 Department of Oral-Maxillofacial Surgery and Orthodontics, The University of Tokyo Hospital, 7-3-1 Hongo, Bunkyo-ku, Tokyo 113-8655 Japan; 6 Center for Information Biology, National Institute of Genetics, 1111 Yata, Mishima, Shizuoka 411-8540 Japan

**Keywords:** Chromatin conformation, Contact domains, Boundary, CTCF, *cis* interaction

## Abstract

**Background:**

Contact domains of chromatin serve as a fundamental unit to regulate action of enhancers for target genes. Looping between a pair of CCCTC-binding factor (CTCF)-binding sites in convergent orientations underlies the formation of contact domains, while those in divergent orientations establish domain boundaries. However, every CTCF site is not necessarily engaged in loop or boundary structures, leaving functions of CTCF in varied genomic contexts still elusive. The locus containing *Tfap2c* and *Bmp7* encompasses two contact domains separated by a region between the two genes, termed transition zone (TZ), characterized by two arrays of CTCF sites in divergent configuration. In this study, we created deletion and inversion alleles of these and other regions across the locus and investigated how they impinge on the conformation.

**Results:**

Deletion of the whole two CTCF arrays with the CRISPR/Cas9 system resulted in impairment of blocking of chromatin contacts by the TZ, as assessed by the circular chromatin conformation capture assay (4C-seq). Deletion and inversion of either of the two arrays similarly, but less pronouncedly, led to reduction in the blocking activity. Thus, the divergent configuration provides the TZ with the strong boundary activity. Uniquely, we show the TZ harbors a 50-kb region within one of the two arrays that contacts broadly with the both flanking intervals, regardless of the presence or orientation of the other CTCF array. Further, we show the boundary CTCF array has little impact on intra-domain folding; instead, locally associating CTCF sites greatly affect it.

**Conclusions:**

Our results show that the TZ not only separates the two domains, but also bears a wide interval that shows isotropic behavior of chromatin folding, indicating a potentially complex nature of actual boundaries in the genome. We also show that CTCF-binding sites inside a domain greatly contribute to the intra-domain folding of chromatin. Thus, the study reveals diverse and context-dependent roles of CTCF in organizing chromatin conformation at different levels.

**Electronic supplementary material:**

The online version of this article (10.1186/s13072-018-0221-1) contains supplementary material, which is available to authorized users.

## Background

The chromatin is highly folded in the nucleus of the eukaryotic cells. A pattern of the chromatin folding either facilitates or prevents interactions between genes and *cis*-regulatory elements such as enhancers. Therefore, control of the chromatin folding is a fundamental step in gene regulation [1]. The genome-wide mappings of chromatin contacts (Hi-C) [2] have revealed that the genome is partitioned into distinct blocks, called topologically associating domains (TADs) or contact domains, within which the genomic regions more preferentially contact with each other than those outside [3–6]. Contact domains restrict allocation of enhancers to target genes within themselves and thus largely define regulatory domains, which are genomic intervals where set of enhancers can pervasively act on genes located inside [7]. Enhancer allocation to target genes following the structural partition of contact domains has been functionally demonstrated at several model loci [8–16]. Genomic rearrangements such as deletion, inversion and duplication involving domain boundaries and enhancers occur naturally in humans and lead to severe genetic diseases due to mis-expression of genes [12, 17, 18].

The underlying mechanism for formation and partitioning of the contact domains is currently explained by the extrusion model, in which cohesin complex plays a major role together with CCCTC-binding factors (CTCF) [19, 20]. According to this model, the cohesin complex is loaded onto the genome and extrudes the chromatin fiber while forming a loop. The extruder is often stabilized at a binding site of CTCF whose binding motif is oriented toward it, but not those orienting the opposite. Thus, a region bound by CTCF predominantly contacts with a genomic interval on the side that it directs through the extrusion loop, exhibiting directionality of chromatin folding. Particularly, the loop is stabilized at a pair of CTCF-binding sites that are in convergent orientation with each other. This looping facilitates contacts among regions inside the looped interval and therefore essentially serves as an entity of contact domains [19, 20].

Though the extrusion model still awaits direct evidence to prove the validity, it well corroborates experimental observations presented so far: bridging via cohesin complexes [8, 21–24] exclusively takes place between a pair of CTCF-binding sites in convergent orientations [6, 25]; CTCF-binding sites, particularly those in divergent orientations, are enriched at boundary regions between contact domains [25, 26]. Further, it was experimentally shown that inversion of CTCF-binding sites leads to alteration in directionality of chromatin folding of the region [19, 27, 28]. Thus, it is widely accepted that the directional folding of chromatin determined by CTCF shapes the higher-order conformation of chromatin through the cohesin extrusion [19, 20].

However, it is still uncertain how various patterns of CTCF bindings on the genome impact on the chromatin conformation. Both in silico modeling and experimental validation showed that a CTCF site orienting one side is sometimes enough to establish a boundary between domains [19]. This is because such a CTCF site forms a stable loop with its pairing CTCF site, which then excludes invasion of an extrusion complex over from the other side of the genomic interval and establishes an exclusion domain [19]. On the other hand, many CTCF-binding sites are rather located within contact domains [4, 19]. Needless to say, not every CTCF site has unique partner of CTCF sites for looping nor is engaged in boundary formation. However, most of the CTCF sites subjected to functional studies so far was those involved in loop formation around domain boundaries [8–10, 19, 24, 27–29]. Therefore, the full spectrum of the CTCF functions in organizing chromatin conformation remains elusive.

**Fig. 1:**
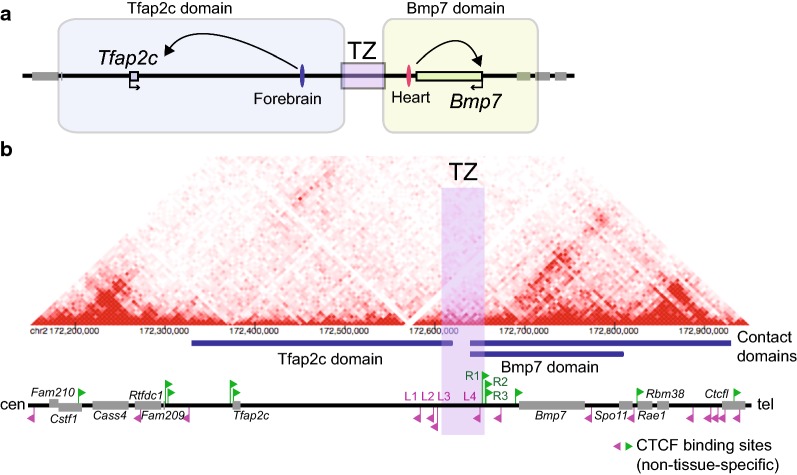
The regulatory and topological domain organization at the *Tfap2c*–*Bmp7* locus of the mouse. **a** The schema of the enhancer regulation at the *Tfap2c*–*Bmp7* locus. The locus consists of two topological domains, separated by the action of the boundary region, TZ, in between. Allocation of the forebrain and heart enhancers to *Tfap2c* and *Bmp7*, respectively, follows the structural partition [11]. **b** Hi-C domains and CTCF bindings at the *Tfap2c*–*Bmp7* locus of the mouse (chr2:172150000-172950000, mm9) genome. The left points to the centromere, while the right points to the telomere. The Hi-C heat maps are generated by the 3D Genome Browser [30] based on the data of CH12 cells at the resolution of 5 kb [6]. Contact domains called from the Hi-C data are depicted by blue bars [6]. Flags along the genomic positions represent non-tissue-specific CTCF-binding sites that are called as peaks in 14 or more out of the 31 ChIP-seq data sets generated from different biological samples by the ENCODE project listed in Additional file 2: Table S1 [32]. The orientation of the binding motifs is indicated by the pointing of the arrowheads as well as their color, with magenta and green indicating leftward and rightward orientations, respectively. As depicted, we named the four centromeric CTCF sites around the TZ as TZ-L1, L2, L3 and L4 and collectively referred to them as TZ-L. Also we named the three telomeric CTCF sites of the TZ as TZ-R1, R2 and R3 and collectively as TZ-R

The two developmental genes *Tfap2c* and *Bmp7* are adjacent with each other, intervened by a ca. 310-kb noncoding region in mice. A previous study showed that the locus is partitioned into two topological domains by action of a discrete region termed transition zone (TZ) [11] (Fig. 1a). This topological partition well corresponds to transition of regulatory domains at the locus in various embryonic tissues examined [11]. The TZ established a domain boundary wherever it was located in the genome upon large inversions that rearranged its surrounding sequences [11]. Enhancers were also reallocated to genes in the domain that they belong to, defined by the TZ, upon the rearrangements [11]. Thus, the locus represents one of several loci where the functional role of chromatin conformation is well described in vivo. However, the precise functional unit of the TZ has not been determined, and mechanism how it separates the topology remained elusive.

In this study, we investigated how the arrangement of CTCF-binding sites around the locus organizes the chromatin conformation. Using CRISPR/Cas9 genome-editing system efficiently, we produced comprehensively deletion and inversion alleles of CTCF-binding sites around the TZ as well as those within a domain that do not constitute domain boundaries in the mouse embryonic stem (ES) cells. The TZ largely consists of two arrays of CTCF-binding sites in divergent orientations, which we show is the more effective configuration in blocking chromatin contacts than those arrayed in one direction. Moreover, we show that directionality by CTCF-binding sites inside a domain is critical for the intra-domain contact pattern. Thus, our results highlight differential contribution of those CTCF sites to the higher-order chromatin conformation of the locus.

## Results

### CTCF bindings and chromatin contact domains at the *Tfap2c*–*Bmp7* locus

The genome-wide chromatin contact map is revealed to 5-kb resolution by Hi-C in the mouse CH12 cell line [6]. From the Hi-C data, two contact domains are called around the *Tfap2c*–*Bmp7* locus, one encompassing *Tfap2c*, and the other *Bmp7*, each hereafter referred to as Tfap2c domain and Bmp7 domain, respectively [6, 30] (Fig. 1b). The domain partition matches the position of the TZ, which was previously identified by 4C-seq (circular chromatin conformation capture assay followed by high-throughput sequencing: detecting DNA fragments contacting with a given region comprehensively [31]) in various embryonic tissues [11]. We examined data of ChIP-seq (chromatin immunoprecipitation followed by high-throughput sequencing) for CTCF binding from 31 different biological samples produced by the ENCODE project [32] (Additional file 2: Table S1). We extracted regions called as CTCF-binding peaks in 14 or more of the data out of the 31 as relatively constant binding sites of CTCF. Then, we analyzed the orientation of the CTCF-binding motif sequences at these peaks using an in silico prediction tools [33, 34] (Fig. 1b). Strikingly, two arrays of CTCF-binding sites are present around the TZ: one consisting of four binding sites orienting toward *Tfap2c*, referred to as TZ-L1, L2, L3, L4 in this order from the centromeric side and collectively as TZ-L, and the other of three sites orienting toward *Bmp7*, referred to as TZ-R1, R2, R3 in this order from the centromeric side and collectively as TZ-R (Fig. 1b). Thus, the two arrays are arranged in divergent configuration just over the TZ, which is the typical hallmark of domain boundaries [26, 27].

### Generation of locally haploid ES cells as the model for efficient mutagenesis

**Fig. 2:**
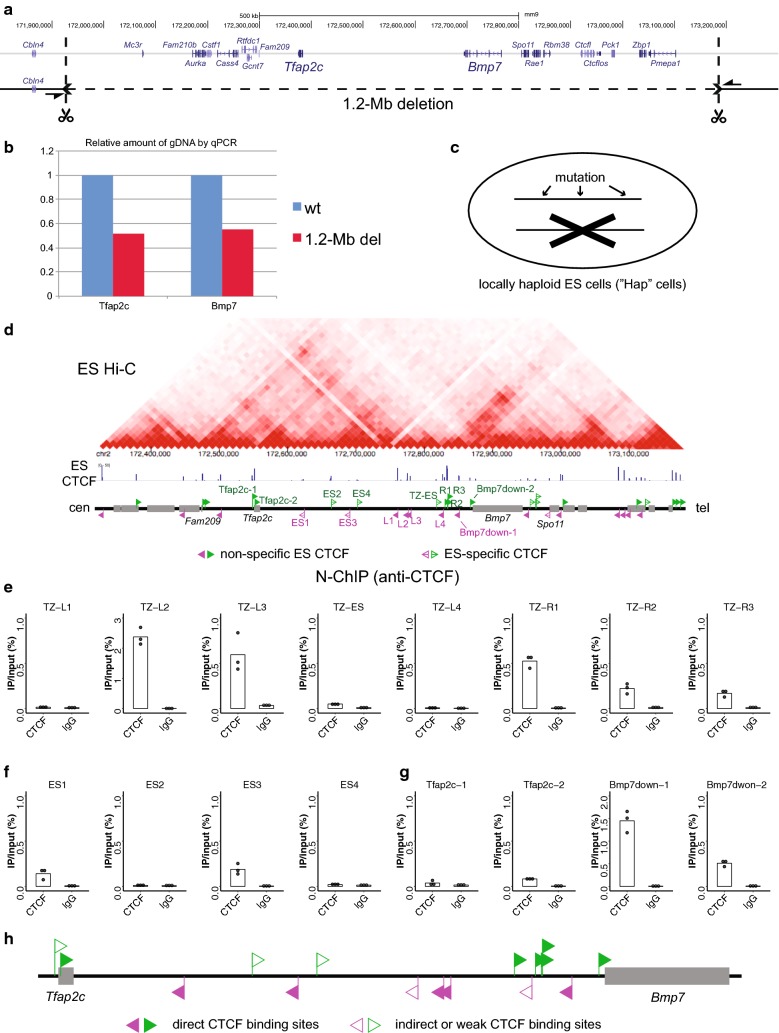
Establishment of a locally haploid ES cell line for efficient mutagenesis assay of the *Tfap2c*–*Bmp7* region. **a** CRISPR targets (represented by scissors) were designed to delete the 1.2-Mb region encompassing the whole locus of *Tfap2c*-*Bmp7* region. The deletion was confirmed by PCR amplification with primers flanking the deleted region (arrows, Additional file 2: Table S4). **b** Confirmation of the local haploidy by qPCR against *Tfap2c* and *Bmp7* genic regions. **c** Strategy to efficiently generate mutations without being bothered by the other masking allele. **d** The Hi-C and CTCF-binding map around the *Tfap2c*-*Bmp7* locus in ES cells. The Hi-C data are from Bonev et al. (2017) [37]. CTCF ChIP-seq peaks called in three independent studies [32, 37, 38] are represented as CTCF-binding sites in ES cells. Those that are not included in the non-tissue-specific sites in Fig. 1b are depicted with arrowheads with a centerline as ES-specific CTCF-binding sites. **e**–**g** Enrichment of indicated regions by N-ChIP with anti-CTCF antibody (left) or normal rabbit IgG control (right). Each dot represents results of independently performed N-ChIP experiments (N = 3), means of which are indicated by bars. **h** The CTCF-binding sites from the upstream of *Tfap2c* to the downstream of *Bmp7* are classified to either direct CTCF-binding sites (filled arrowheads) or indirect/weak CTCF-binding sites (open arrowheads) according to the results of the N-ChIP-qPCR (**e**–**g**)

The configuration suggests that the two CTCF arrays in divergent orientations at the TZ are responsible for the structural partitioning. To test this, we serially performed targeted mutagenesis around the locus, with CTCF-binding sites as landmarks, in the mouse ES cells. As the diploid nature of the cells demands simultaneous mutations of the two alleles to assess the functionality, we first deleted one of the two alleles by 1.2 megabase (Mb) encompassing the whole locus with the CRISPR/Cas9 system targeting the both ends of the 1.2-Mb region as described before (Fig. 2a) [35, 36]. We confirmed the deletion by PCR amplification with primer pairs sandwiching the deleted region (Fig. 2a, Additional file 2: Table S4), as well as by qPCR showing the local haploidy (Fig. 2b). With this clone, termed “Hap,” we now only need to mutate one allele to test the functionality of the genomic elements within the region (Fig. 2c).

The Hi-C data in ES cells illustrate separation of domains at the TZ [4, 11, 37] (Fig. 2d). We note several ES-specific binding sites of CTCF around the locus. Although these bindings seem relatively weak as indicated by the height of the peaks (Fig. 2d), some are consistently detected by independent ChIP-seq experiments [32, 37, 38]. Therefore, we re-extracted CTCF-binding sites that were called in all of recent three studies in ES cells [32, 37, 38], and determined the orientation (Fig. 2d). All of the non-tissue-specific CTCF-binding sites around the locus (Fig. 1b) are included in the list of ES-CTCF-binding sites, except the one located between *Rbm38* and *Ctcfl*. In addition, there are five ES-specific CTCF-binding sites: four between *Tfap2c* and TZ-L1, referred to as ES1, 2, 3, 4 in this order from the centromeric side; the other located between TZ-L3 and TZ-L4, referred to as TZ-ES (Fig. 2d).

### N-ChIP assay to distinguish direct CTCF-binding sites from indirect/weak ones

The CTCF-binding motifs and their orientations were only determined by in silico prediction. Therefore, it is not entirely certain whether the identified motifs are actually recognized by CTCF within the sites or not. Normally, ChIP experiments are performed after cross-linking. This leads to detection of binding sites that are only indirectly associated with CTCF through formation of specific loops with another directly binding site [39]. Recent studies in fact indicated that loops are also formed by non-CTCF proteins [40, 41]. Such indirect binding seems predominant in the genome, as quite a few number of CTCF-binding sites lack the canonical motif [42].

In order to grasp how reliable the called motifs are, we performed native chromatin immunoprecipitation followed by qPCR assays (N-ChIP-qPCR) in the “Hap” ES cells. Since N-ChIP only detects direct binding sites [39], this should tell confidence of the motif determination. TZ-L2 and L3 of the TZ-L array, as well as all the three sites of the TZ-R, were robustly enriched by N-ChIP, showing they are in fact directly bound by CTCF (Fig. 2e). However, the TZ-L1 and L4 were not detected (Fig. 2e), indicating that the CTCF binding at these sites is only indirect or weak. The ES-specific site TZ-ES was slightly enriched (Fig. 2e). We further tested enrichment of sites around *Tfap2c* (termed Tfap2c-1 and 2; see Fig. 2d) and those between TZ-R3 and *Bmp7* (Bmp7down-1 and 2; see Fig. 2d), and other ES-specific binding sites, ES1, 2, 3, 4, by N-ChIP (Fig. 2f, g). The results show that Tfap2c-2, Bmp7down-1, 2, ES1 and ES3 are directly recognized by CTCF (Fig. 2f, g). However, the other Tfap2c-1, ES2 and ES4 sites were not enriched by the N-ChIP (Fig. 2f, g). Thus, the called motifs and their orientations were assured at the sites where direct binding of CTCF was confirmed by N-ChIP. On the other hand, we should remain cautious about the motifs determined at the other sites that lack evidence of direct recognition by CTCF (Fig. 2h).

### Deletion of the divergent CTCF arrays led to invasion of chromatin contacts

The TZ was first identified as a genomic region that autonomously partitions chromatin topology upon translocation to different genomic contexts by artificially introduced inversion [11]. In this sense, its functional unit was only narrowed down to the interval of the smallest inversion tested (i.e., chr2:172556092-172689701 in mm9) [11], which includes both the TZ-L and R arrays and the Bmp7down-1 and 2 sites.

**Fig. 3:**
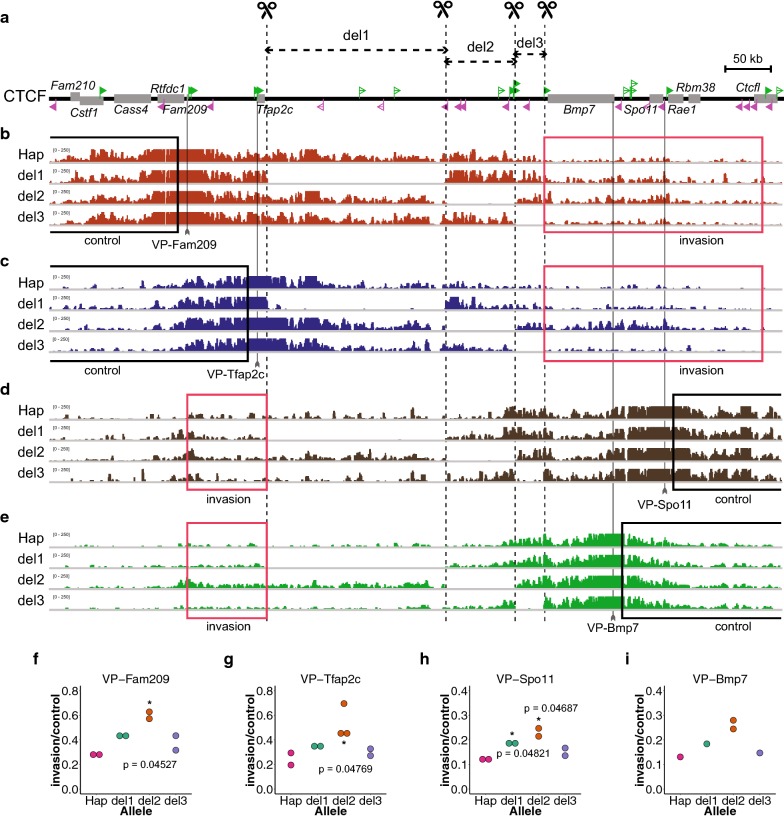
Deletion of the two CTCF-binding arrays in divergent orientations led to increase in inter-domain contacts. **a** We produced the three deletion alleles termed del1, del2 and del3, as depicted, which consecutively cover the whole intergenic region between *Tfap2c* and *Bmp7*. Note that del2 completely diminishes the two arrays of CTCF sites, TZ-L and TZ-R, while the other two delete the adjacent regions. The CTCF-binding sites in ES cells are depicted as in Fig. 2d. **b**–**e** 4C-seq plots from the VP-Fam209 (**b**), VP-Tfap2c (**c**), VP-Spo11 (**d**) and VP-Bmp7 (**e**). **f**–**i** The ratios of “invasion reads” to “control reads,” each mapped to the areas depicted by the red and black rectangles, respectively, in (**b**–**e**), were compared between different alleles, for each viewpoint. Each dot represents a replicate of differently prepared 4C-seq libraries from collection of cells. * indicates p < 0.05 by one-sided permutation test against the Hap allele. Note that del2 allowed the inter-domain contacts most among the three deletions for all of the viewpoints, although the size of the deletion is smaller than that of del1

To understand the functional relevance of TZ-L and R, and other regions to the structural partition by the TZ, we first produced three consecutive deletion alleles spanning the whole intergenic region between *Tfap2c* and *Bmp7* of the Hap clone: One is the deletion of the two CTCF arrays, TZ-L and TZ-R (del2), the others deleting the intervals either between *Tfap2c* and TZ-L (del1) or between TZ-R and *Bmp7* (del3) (Fig. 3a). We performed 4C-seq to compare the chromatin conformation between the wild-type allele of the Hap clone and the three deletion alleles (Fig. 3).

*Fam209* and *Spo11* are located near the borders of the Tfap2c and Bmp7 domains, respectively, both harboring CTCF-binding sites in the vicinity (Fig. 1b). In the wild-type allele, the detected contacts of the viewpoints of *Fam209* (VP-Fam209) and *Spo11* (VP-Spo11) well extended to the intergenic region between *Tfap2c* and *Bmp7*, but mostly up to the TZ (Fig. 3b, d, and Additional file 1: Figure S1). Similarly, from the viewpoint around the transcription start sites of *Tfap2c* (VP-Tfap2c) and *Bmp7* (VP-Bmp7), the contact barely extends beyond the TZ to the neighboring domain (Fig. 3c, e and Additional file 1: Figure S1). We then compared the profiles with those in the deletion alleles. Apparently the del2 resulted in extensive inter-domain contacts beyond the TZ, though the other deletion did not to that extent (Fig. 3b–e, and Additional file 1: Figure S1).

To quantitatively compare these inter-domain contacts, we counted the number of reads mapped to a defined region in the adjacent domain beyond the TZ as “invasion reads” (red rectangles in Fig. 3b–e). We also counted the reads mapped to an interval lying on the opposite side of the TZ from the viewpoint up to the end of the locally haploid region (i.e., the 1.2-Mb deletion region) as “control reads,” which we assumed to be little affected by the deletions. Then, we simply divided the number of “invasion reads” by that of the “control reads” as an indicator of inter-domain contacts of the viewpoints beyond the TZ (Fig. 3f–i). Since the indicator relies only on the counts over regions that were subjected to none of the three deletions, this should be well comparable among the different alleles.

Strikingly, the del2 allele always showed the largest rate of invasion for all the four viewpoints (Fig. 3f–i). Such increase may be partly due to the decrease in the genomic distance to the next domain. However, the size of the deletion was largest with the del1, which showed less degree of invasion than the del2. Therefore, the increase in invasion in del2 is not merely due to the distance effect, but due to the loss of functional elements, most likely the arrays of the CTCF-binding sites.

### The divergent configuration was the most effective in blocking chromatin contacts

**Fig. 4:**
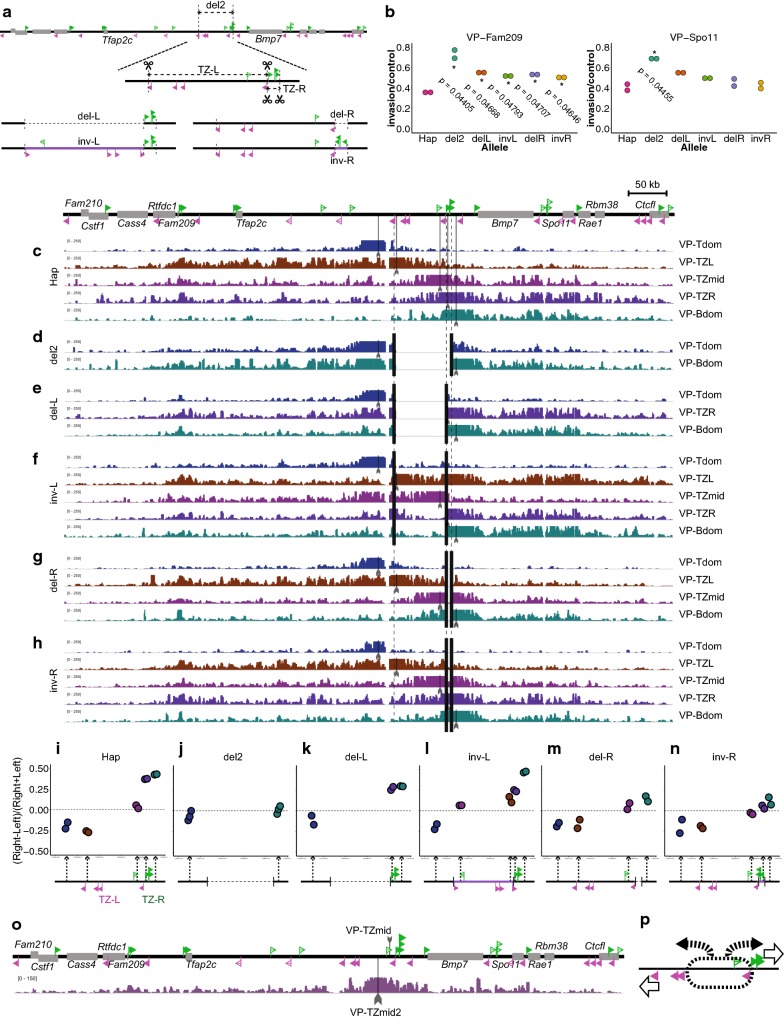
Deletion and inversion of each one of the two CTCF arrays at the TZ. **a** CRISPR targets were designed as depicted, to produce deletion and inversion of either centromeric (TZ-L) or telomeric (TZ-R) of the TZ CTCF arrays. **b** Comparison of the inter-domain contacts (ratio of the invasion reads to the control reads; see Additional file 1: Figure S2) of 4C-seq from the VP-Fam209 and VP-Spo11. * indicates p < 0.05 by one-sided permutation test against the Hap allele. **c**–**h** 4C-seq plots of the Hap cells (**c**), del2 (**d**), del-L (**e**), inv-L (**f**), del-R (**g**) and inv-R (**h**) from the viewpoints around the TZ region. The viewpoints are depicted under each plot. They were VP-Tdom to the left of the TZ-L, VP-TZL at the TZ-L, VP-TZmid between TZ-L3 and TZ-L4, VP-TZR at the TZ-R, and the VP-Bdom to the right of the TZ-R. **i**–**n** Directionality scores of chromatin folding of the viewpoints are plotted for each allele configuration. Directionality score was determined as the difference of the number of mapped reads between the left and right intervals within the 200-kb distance from the viewpoint, which was normalized by the sum of them. **o** The 4C-seq plot of wild-type ES cells from a viewpoint between TZ-L3 and VP-TZmid (VP-TZmid2). **p** The schema of the folding property of the TZ. The TZ-L and the TZ-R together generate the diverging directionality of the chromatin folding, thus establishing a boundary. However, the region between the TZ-L3 and TZ-L4 rather contacts with the both sides equivalently

To explore how the CTCF arrays impinge on the structural partitioning of the chromatin at the TZ, the TZ-L and TZ-R were each deleted (del-L and del-R) and inverted (inv-L and inv-R), respectively (Fig. 4a). Strikingly, the 4C-seq from the VP-Fam209 detected significantly more degree of inter-domain contacts in all of the four deletion/inversion alleles than the wild-type allele (Fig. 4b and Additional file 1: Figure S2). Those from the VP-Spo11 also showed similarly increased invasion rates in the rearranged alleles, though statistical significance was not called (Fig. 4b and Additional file 1: Figure S2). The results suggest that these configurations, in which the CTCF-binding sites, except the weak TZ-ES, are arranged in one direction, are less effective in structural partitioning than the divergent configuration of the wild-type allele. However, their inter-domain contacts were never as much as those in the del2, indicating that a cluster of CTCF sites orienting one direction is able to block the contact to some extent (Fig. 4b).

To understand how the chromatin conformation is organized in the different configurations of the CTCF-binding sites, we further performed 4C-seq from five viewpoints around the TZ: VP-Tdom to the centromeric side of the TZ-L within the Tfap2c domain; VP-TZL between the TZ-L1 and L2; VP-TZmid at the middle of the TZ near the TZ-L4; VP-TZR between the TZ-R1 and R2; VP-Bdom to the telomeric side of the TZ-R in the Bmp7 domain (Fig. 4c–h). In the wild-type allele of the Hap cells, the contacts of the VP-Tdom and VP-TZL are biased toward the centromeric side, while those of the VP-Bdom and VP-TZR are more toward the telomeric side. The contact profile of the VP-TZmid appeared isotropic in both directions (Fig. 4c). We scored the directionality of contacts from a viewpoint by calculating the difference of read counts between those mapped centromeric and telomeric within the 200-kb distance, and normalizing it by the sum of them (Fig. 4i–n). The rational that we took the 200-kb region into account is that this is roughly the maximum distance from the viewpoints that does not exceed the edge of the domains on the both sides of the TZ. In the Hap cells, the centromeric (VP-Tdom and VP-TZL) and telomeric (VP-Bdom and VP-TZR) viewpoints exhibited the directionality of minus and plus values, i.e., directing more toward the centromere and telomere, respectively, while the directionality of VP-TZmid was near zero. Thus, these plots clearly showed that the diverging directionality of chromatin folding is established at the TZ (Fig. 4c, i).

In the del2 allele, the contact of VP-Tdom and VP-Bdom well extended to the adjacent Bmp7 and Tfap2c domains, respectively (Fig. 4d), and the directionality of chromatin folding almost disappeared (Fig. 4j). This indicates that the other CTCF-binding sites flanking the TZ (i.e., ES1, 2, 3 and 4, Bmp7down-1 and 2) do not contribute much to the diverging chromatin folding. Thus, the results further argue that the two CTCF arrays indeed establish the boundary. Interestingly, the directionality profiles around the TZ in the del- and inv-L and del- and inv-R alleles were altered in different ways with each other (Fig. 4e–h, k–n). The del-L still maintained the directionality, but the degree was weaker than the wild type, indicating that an array of CTCF in one direction (i.e., TZ-R) is able to make a weak boundary (Fig. 4e, k). The inv-L maintained the directionality rather strongly at VP-Tdom and VP-Bdom (Fig. 4f, l). However, the directionality at the CTCF sites (VP-TZL and VP-TZR) was not as strong as in the wild type, which may be reflected to the weak extension of the inter-domain contacts from VP-Fam209 and VP-Spo11 (Fig. 4b, l). This inversion allele arranges the six binding sites of CTCF (TZ-L3, L2, L1, R1, R2, R3) in a narrow region at the telomeric side of the TZ in one direction toward the telomere (see Fig. 4l). This redundancy may have resulted in loss of the directionality at these sites. Particularly, those at the more centromeric side, which are located outside from the extrusion complex coming from the *Bmp7* side, showed lower degree of directionality (Fig. 4l). Also, the absence of the diverging CTCF sites toward the centromere, which should have prevented invasion of an extrusion complex from the centromeric side to the TZ-R, may have affected the decrease in the directionality at VP-TZR in both the del- and inv-L alleles (Fig. 4k, l).

The del-R allele similarly showed reduction in the directionality bias particularly at the telomeric side (VP-Bdom), although certain degree of directionality was still maintained (Fig. 4g, m). Interestingly, the inversion of the same region (inv-R), which adds to the del-R three CTCF-binding sites in the same orientation toward centromere, did not result in higher directionality than the del-R (Fig. 4n). This could be again accounted by the redundancy of the CTCF sites, which may discharge those located outside from the extrusion complex.

It should be noted that in all the mutant configurations as well as the wild-type allele, the contact profiles from the TZmid were equally distributed toward both centromeric and telomeric sides (Fig. 4i, l–n). Such isotropy from the same viewpoint fragment was also described in the previous study (see Fig. 3e in Tsujimura et al. 2015 [11]). The present results show that this isotropy is not dependent on the TZ-R CTCF array. Instead, bias of directionality at VP-Tdom in inv-L and that at VP-Bdom in del-R might suggest that the presence of the TZ-L4 plays a role (Fig. 4l, m). On the other hand, 4C-seq from a viewpoint between TZ-L3 and TZ-ES (VP-TZmid2) showed also isotropic contact profile of the region in the wild-type ES cells (Fig. 4o). Therefore, the TZ-ES site does not seem to contribute much to this. Although the mechanism remains elusive, the profiles show that the TZ possesses a broad region that robustly exhibits unbiased contact with the both directions, regardless of the presence or orientation of the telomeric CTCF array TZ-R (Fig. 4p).

### The folding directionality is mostly determined locally

**Fig. 5:**
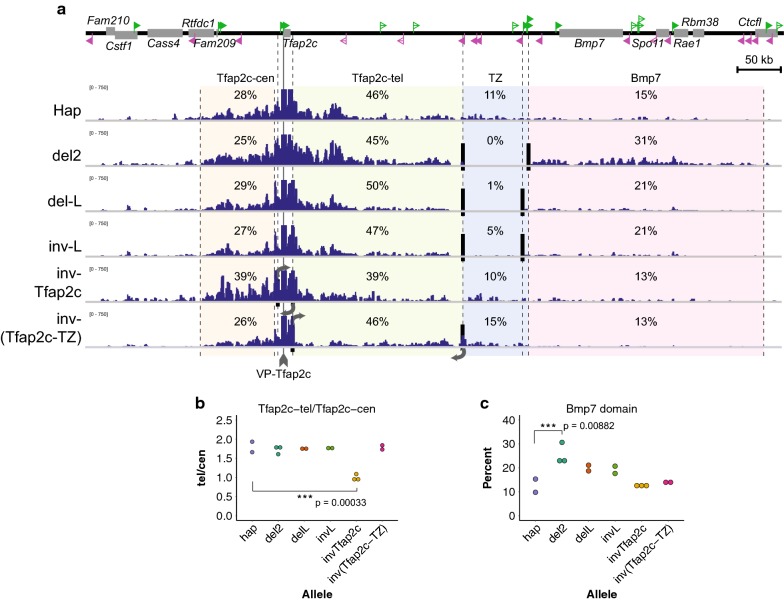
Non-boundary CTCF for organizing intra-domain chromatin folding. **a** The 4C-seq profiles from the VP-Tfap2c of different deletion and inversion alleles. The locus is divided into four areas (Tfap2c-cen, Tfap2c-tel, TZ, Bmp7 domain), and percentages of reads mapped among them are shown. **b** The ratios of read numbers in Tfap2c-tel to those in Tfap2c-cen are depicted for each allele, to compare the directionality of intra-domain chromatin folding. Each plot represents independent replicates. **c** The percentage of reads within the Bmp7 domain is plotted for the different 4C-seq results and compared between different alleles. *** indicates p < 0.001 by one-way ANOVA (analysis of variance), followed by Tukey’s multiple-comparison post hoc test. The statistical significance against the Hap allele is indicated

As the CTCF array TZ-L pervasively contacts with the interval within the Tfap2c domain (Fig. 4c), it is plausible that the array may also impact on the folding directionality of a region inside the domain by sequestering it. Particularly, *Tfap2c*, which harbors CTCF binding toward the telomeric (i.e., TZ and *Bmp7*) side, may form stable loops with TZ-L. Therefore, the folding directionality of *Tfap2c* might be affected by the presence of TZ-L. To test this, we compared the contact distribution of VP-Tfap2c between the mutant alleles around the TZ (Fig. 5). To represent the distribution of the contacting regions, we defined four intervals, centromeric (Tfap2c-cen) and telomeric (Tfap2c-tel) parts of the Tfap2c domain, TZ and the Bmp7 domain, and showed percentage of the total counts mapped to these intervals (Fig. 5a). In the wild-type allele, almost a quarter and half of the reads were mapped to Tfap2c-cen and Tfap2c-tel, respectively. Roughly halves of the rest were mapped to the TZ and the Bmp7 domain, respectively (Fig. 5a). Intriguingly, the proportions of the intra-domain contacts (i.e., Tfap2c-cen and Tfap2c-tel) were largely unchanged by the deletion of the TZ (del2), or by the deletion and inversion of TZ-L (del-L and inv-L) (Fig. 5a, b). In the del2 allele, the contacts with the TZ region in the wild-type allele appeared to be only re-distributed to the Bmp7 domain without impacting on the contacts with the Tfap2c-cen and the Tfap2c-tel zones (Fig. 5a, c). The deletion and inversion of the TZ-L also only led to a decrease and increase in reads on the TZ and the Bmp7 domain, respectively, without much affecting the proportion within the Tfap2c domain (Fig. 5a–c). These results suggest that the presence of the CTCF array, TZ-L, orienting to *Tfap2c* has little impact on the folding directionality of VP-Tfap2c. Instead, the CTCF array only blocks extension of chromatin folding from *Tfap2c* into the Bmp7 domain.

On the other hand, inversion of *Tfap2*c (inv-Tfap2c), which together altered the orientation of the two associating CTCF sites, greatly and significantly increased the proportion of the Tfap2c-cen reads over the Tfap2c-tel reads (Fig. 5a, b). We further produced an inversion allele of the region between *Tfap2c* and the TZ, inv-(Tfap2c-TZ), and performed the 4C-seq. Although this strongly affected the contact profile within the inverted region (Fig. 5a), again the ratio between the centromeric and telomeric side within the Tfap2c domain was unchanged (Fig. 5a, b). These results strongly indicate that the intra-domain folding directionality of a region is more determined by locally associating *cis*-elements such as CTCF-binding sites than by those located further around the domain edge, as long as the distance of a given domain is same.

## Discussion

The three-dimensional chromatin organization is a fundamental entity for gene regulation, as it mediates communications between genes and *cis*-regulatory elements. The extrusion mechanism by CTCF and cohesin complexes explains well the principle of formation of contact domains through looping between a pair of CTCF-binding sites in convergent orientations. As included in the model, a pair of CTCF-binding sites in divergent orientations is enriched at boundaries between contact domains [25, 26]. However, in silico prediction and experimental assays showed that one direction of CTCF is also enough to establish loop and exclusion domains as a boundary at the both flanking sides, though such configuration is less likely to be called as so [19]. Thus, how an arrangement of CTCF sites impacts on the chromatin conformation at different loci needs to be studied in details.

In this study, we characterized how the CTCF-binding sites contribute to the chromatin conformation at the *Tfap2c*-*Bmp7* locus. Previously, it was clearly shown that a discrete region termed TZ autonomously establishes a structural boundary and allocates enhancers to target genes [11]. However, the precise functional unit of the TZ was not defined and the mechanism of the structural partitioning remained totally elusive. Here, we found that the TZ is in fact characterized by two arrays of CTCF-binding sites in divergent orientations with each other (Fig. 1b). In ES cells, TZ-ES is present within the TZ-L array as an ES-specific CTCF-binding site orienting toward telomere, the opposite direction from the other sites of the array. We think contribution of the TZ-ES site to the chromatin conformation is quite limited. Firstly, the structural partition at the TZ is tissue invariant, indicating this should be achieved more by tissue-invariant factors. Secondly, our N-ChIP and other ChIP-seq signals show only a little binding of CTCF there (Fig. 2d, e).

**Fig. 6:**
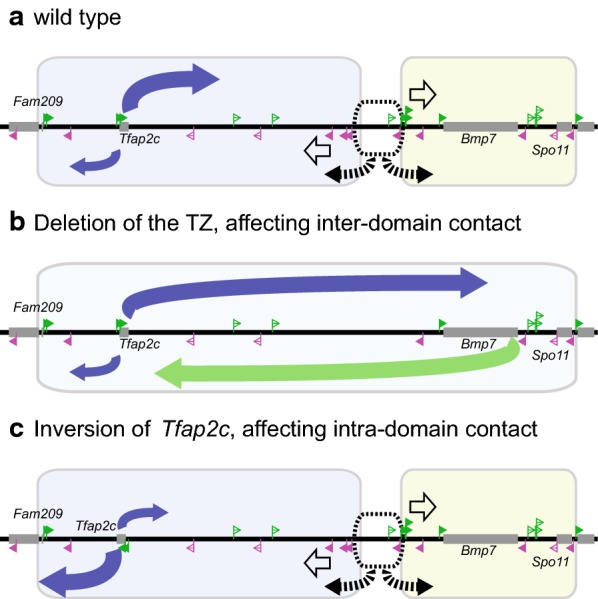
Directionality control of chromatin folding at the *Tfap2c*–*Bmp7* locus. **a** In the normal wild-type configuration, the locus is partitioned into the two domains by the action of the TZ. This ability originates from the divergent configuration of the two CTCF arrays, which induces directional chromatin folding to the diverging orientations from the TZ. Note that the central region of ca. 50 kb of the TZ behaves isotropically in terms of chromatin folding, which provides the TZ with another feature than blocking of the contact domains. **b** The directional folding disappeared upon the deletion of the TZ, which resulted in a merge of chromatin domains. **c** The folding directionality of *Tfap2c* is greatly influenced by the CTCF binding in the vicinity, but little by those at the TZ. The inversion of *Tfap2c* together with the CTCF sites altered the directionality drastically

The deletion of the whole of TZ-L and R resulted in extension of the chromatin contact to neighboring domains as well as loss of the divergent directionality of chromatin folding around the TZ, showing that the region is indeed responsible for the partitioning (Figs. 3, 4, 6). We further produced deletions and inversions of each one of the two CTCF arrays to challenge the divergent configuration (Fig. 4). Then, an array in one direction was enough to block chromatin contacts and to produce folding directionality more than the del2 allele lacking the whole set of the two arrays, corroborating the formation of loop and exclusion domains [19]. However, the degree of conformational separation with these configurations never reached that of the wild type. This held true even when the total number of the CTCF-binding sites was maintained by the inversions. Thus, the results show that the divergent configuration is the pattern that most strongly establishes a domain boundary (Fig. 6). The leaky contacts through the CTCF-binding sites arrayed in one direction might be explained by invasion of extrusion complexes from behind the CTCF arrays, that is, the side of the exclusion domain. A recent study suggested that cohesin loops are dynamically released by WAPL [43]. Therefore, there should be certain window of time when the CTCF sites are not engaged in loop formation and do not prevent invasion of extrusion complexes from the opposite side. However, in case the CTCF-binding sites are arranged in divergent configuration, the extrusion complex should be more likely paused by CTCF orienting toward it.

We note that the divergent configuration is well conserved at the domain boundary in humans (Additional file 1: Figure S3a), which further supports the notion that this is critical for the gene regulation of the locus. Particularly, it should be emphasized that both of the two CTCF arrays at the TZ are composed of not a single binding site but multiple ones. Though functional roles of this redundancy need to be tested further, it may help to enforce the partitioning between the two domains. Effective blocking of enhancer activity by redundant CTCF binding was indirectly suggested in a previous report [44].

Importantly, the N-ChIP-qPCR assay missed enrichment at TZ-L1 and L4 (Fig. 2e). The results may indicate that CTCF only indirectly binds to these sites, or alternatively that the bindings are direct but too weak to be recovered by the N-ChIP without cross-linking. Recent studies showed involvement of other zinc-finger proteins, such as YY1 and ZFN143, in architectural organization of chromatin similarly as CTCF [40, 41, 45]. Therefore, it is possible that these and other unidentified architectural proteins bind to the “indirect” sites and recruit CTCF through loop formation. Even in this scenario, the TZ-R is marked by the three direct binding sites of CTCF. Further, the TZ-L still mainly consists of the two CTCF sites, TZ-L2 and L3, orienting toward centromere, in addition to the weak TZ-ES site. Therefore, the interpretation above should not change much. However, introducing point mutations of the core CTCF-binding motifs only are necessary to exactly determine the role of CTCF. How and to what extent each CTCF-binding site contributes to the structural partition at the TZ needs to be carefully studied in future.

Uniquely, the TZ consists of not just two arrays of CTCF clusters generating divergent directionality of chromatin folding but also a substantially large region between the TZ-L3 and L4 that exhibits isotropic contacts with the both flanking domains (Fig. 4p). The feature of the isotropic folding is quite robust and not dependent on the telomeric CTCF array, TZ-R (Fig. 4i–n). Although what provides the region with the feature remains elusive, the presence of TZ-L4 seems to be the key. Since the N-ChIP failed to show direct binding of CTCF to the TZ-L4, it is required to find out which factors recognize and bind to the region to understand the mechanism.

The impact of possessing such an isotropic region also remains elusive. However, the arrangement is very similar between humans and mice, and there are some conserved sequences inside the region (Additional file 1: Figure S3b), suggesting that the configuration and elements inside should have critical roles. Currently, a boundary region is merely considered as a blocker of chromatin contacts. However, our results, together with future studies on functional roles of the 50-kb region in gene regulation, should highlight that a boundary may possess an additional feature beyond blocking chromatin contacts to distribute genetic information around the locus.

We also asked how the distribution of intra-domain contacts is determined. Intriguingly, the mutations around the TZ did not impact on the folding directionality of *Tfap2c*. This result indicates that loops bridging CTCF sites are not stable enough to solidly sequester chromatin conformation, which is again consistent with the dynamic turnover of the cohesin complex at chromatin by WAPL [43]. To the contrary, inv-Tfap2c greatly influenced on the *Tfap2c* folding. Thus, the folding directionality is determined more locally than globally (Figs. 5, 6). Since Tfap2c-1 is indirect or weak binding site of CTCF, other mechanisms, such as YY1-mediated interaction [40], are not entirely excluded from the control of the directionality. However, the other CTCF-binding sites, Tfap2c-2, is a direct one, and this binding is conserved between mice and humans (Additional file 1: Figure S3a). Therefore, the binding of CTCF should be the primary determinant of the directionality of the intra-domain chromatin folding. Interestingly, a previous study showed that species-specific binding of CTCF inside contact domains contributes to reorganization of intra-domain structures [25]. Investigating into the impact of these intra-domain CTCF sites on gene regulation and evolution should be intriguing. The forebrain enhancer for *Tfap2c* is located at the telomeric side within the domain near the TZ (Fig. 1a) [11]. Further, many unidentified enhancers should be embedded around the locus for *Tfap2c* expression in different tissues. It should be interesting to know how the inversion of *Tfap2c* might affect the gene activation by the enhancers in the forebrain and other tissues in vivo.

Interestingly, the altered directionality of the intra-domain chromatin folding of inv-Tfap2c had only limited impacts on the inter-domain contacts of *Tfap2c* with the Bmp7 domain (Fig. 5a, c). This might suggest that the inter-domain contacts beyond the TZ take place largely due to the physical proximity. Similarly, at the *Hoxd* locus, some inter-domain contacts seem to appear based on the physical distance between them, independently of the domain structure [13]. CTCF/cohesin-independent association of chromatin has been implicated in various systems, particularly between regions of same epigenetic states [3, 38, 46–53]. Such association may also underlie contacts between distinct domains. A previous study in fact demonstrated competing interaction between *Tfap2c* and *Bmp7* across the TZ in the forebrain [11]. Similarly, antagonistic regulation between two adjacent domains was described at the *Hoxd* locus during the limb development [54, 55]. The recent proposal of gene regulation by phase separation might be possibly an interesting model to understand the interaction between the adjacent domains [56]. The effect of being proximal beyond a domain boundary on gene regulation has been dismissed so far. Further studies will be required to gain insight into it.

## Conclusions

The present study showed that the TZ not only separates the two domains, but also bears a wide interval that shows isotropic behavior of chromatin folding. Furthermore, we show that CTCF-binding sites inside a domain greatly contribute to the intra-domain folding of chromatin. These findings suggest that formation of contact domains and blocking of enhancer activity only represent a part of the outcomes of the function of CTCF and other architectural proteins in gene regulation. In this sense, digging into hidden aspects of genome architectures apart from the domain organization will be required to fully understand the gene regulation by chromatin folding.

## Methods

### Analysis of publically available data of CTCF bindings and Hi-C

We downloaded from the ENCODE database [32] BED files listing CTCF-binding peaks (conservative IDR thresholded peaks) detected by ChIP-seq of 31 different biological samples (listed in Additional file 2: Table S1). We first merged the files into one BED file using BEDTools (version 2.26.0) [57] to have lists of all the peaks detected by the experiments. Using this as a query, we counted how many times these peaks are called out of the 31 experiments, with BEDTools. Then, we extracted only the peaks that were called for 14 or more times (more than 45%). Similarly, we obtained three of publically available lists of CTCF-binding sites in mouse ES cells as bed files [32, 37, 38] and extracted those that are consistently called in the three as ES-CTCF-binding sites in the same way as above. To determine the orientation, we input the central 200-bp regions of the peaks to the CTCFBS Prediction Tool [33]. We adopted the outputs based on the motif position weight matrices (PWMs), REN_20 [58], MIT_LM2, MIT_LM7 and MIT_LM23 [59], which basically returned consistent results with each other. We further scanned motifs in the ES-CTCF-binding sites included in the bed file above using GimmeMotifs [34] with PWM from the HOCOMOCO database [60], and confirmed the orientations. We downloaded a bigwig file of the ChIP-seq of CTCF in mouse ES cells from the ENCODE database (file accession: ENCFF069PTO) to visualize it [32]. The CTCF-binding sites and their orientations in human GM12878 cells were retrieved from a previous literature [6].

The Hi-C data and list of contact domains in mouse CH12 cells and human GM12878 cells are from Rao et al. 2014 [6]. The Hi-C in mouse ES cells is from Bonev et al. 2017 [37]. To visualize the Hi-C data, we used the 3D Genome Browser [30].

### Cell culture and CRISPR genome editing

The male mouse ES cell line, B6J-S1^*UTR*^ [61], was kindly provided by RIKEN BRC through the National Bio-Resource Project of the MEXT, Japan. The culture medium was DMEM (SIGMA-ALDRICH, Cat. D5796) containing 0.1 mM 2-mercaptoethanol (Sigma, Cat. M7522), leukemia inhibitory factor (Wako, Cat. 129-05601), penicillin–streptomycin–glutamine (Thermo Fisher Scientific, Cat. 10378-016), nonessential amino acids (Thermo Fisher Scientific, Cat. 11140-050) and 20% knockout serum replacement (Thermo Fisher Scientific, Cat. 10828-028). We cultured the cells on the SNL feeder cells to maintain and on dish coated with thin layer of Matrigel (Corning, Cat. 354277) without feeder cells to expand for use for the 4C-seq and N-ChIP assays.

To perform the genome editing, we cloned the target sequences of CRISPR into the cloning site of pSpCas9(BB)-2A-Puro (PX459), which was gifted from Dr. Feng Zhang (Addgene plasmid # 48139), with *Bbs*I restriction enzyme [62]. The CRISPR target sequences and Oligo DNAs used to integrate the target sequence into the vector are listed in Additional file 2: Table S2. We then transfected the ES cells with the plasmids using Lipofectamine 2000 Transfection Reagent (Thermo Fisher Scientific, Cat. 11668030). We exposed the cells to puromycin (0.5 mg/L) for 2 days from the next day of transfection to enrich positive cells. We always used a pair of plasmids targeting two genomic sites of the locus in a transfection reaction, which should typically result in either deletion or inversion of the interval between them [35, 36], as listed in Additional file 2: Table S3 and depicted in Additional file 1: Figure S4. To isolate positive clones, we spread the transfected cells sparsely (50/cm^2^), picked up the grown colonies and propagated them. To identify correctly edited clones, we performed PCR against the genomic DNA extracted from each clone using primer sets in Additional file 2: Table S4. To confirm haploidy of the locus in the Hap cell line, we quantified the allelic representation of the *Tfap2c* and *Bmp7* genic regions by qPCR, normalized the values with that of *Gapdh* and compared it to normal diploid wild-type cells. The primers used for the qPCR are listed in Additional file 2: Table S5. Positive clones were then propagated for the following 4C-seq assays.

### N-ChIP-qPCR

We basically followed the protocol of N-ChIP for CTCF binding that was developed and described in a previous report [39]. After harvested, cells were resuspended in ChIP dilution buffer (20 mM Tris–HCl pH 8.0, 150 mM NaCl, 2 mM EDTA, 1% Triton X-100), supplemented with 0.05% SDS, 3 mM CaCl_2_ and protease inhibitors, placed on ice for 10 min and briefly incubated at 37 °C for 2 min. Then, 0.3 μl of micrococcal nuclease (NEB, Cat. M0247S) was added per 1.5 million cells. After incubation at 37 °C for 10 min, EDTA and EGTA were added to the final concentrations of 10 mM and 20 mM, respectively, to stop the digestion reaction. The chromatin was solubilized by sonication using Ultrasonic Homogenizer UH-50 (SMT Co., Ltd.) and incubated at 4 °C for 1 h. The cell debris was pelleted and removed by centrifugation. 3.6 μl of anti-CTCF antibody (Millipore, 07-729), or 5 μg of normal rabbit IgG control (Wako, 148-09551) was then incubated with solubilized chromatin from the 1.5 million cells. After 4 h of incubation, the chromatin with the antibodies was incubated with 20 μl of Dynabeads Protein G (Thermo Fisher Scientific, Cat. 10003D) for 1 h. Then, the beads were washed for five times with ChIP dilution buffer supplemented with 0.05% SDS. The immunoprecipitated chromatin was treated with RNaseA (50 ng/μl) at 37 °C for 15 min and then with Proteinase K (100 ng/μl) at 55 °C for 1 h in ChIP extraction buffer (20 mM Tris–HCl pH 8.0, 300 mM NaCl, 10 mM EDTA, 5 mM EGTA, 0.1% SDS). After removal of beads, the DNA was precipitated with ethanol and eluted in 10 mM Tris–HCl pH 8.0. Then, enrichment of DNA was quantified by qPCR. The primers used are listed in Additional file 2: Table S5. Three independent ChIP experiments were performed for both anti-CTCF antibody and IgG control. Data from all the three experiments and their mean values were plotted using the ggplot2 package in R.

### Library prep for 4C-seq and high-throughput sequencing of the libraries

To prepare a 4C-seq library, we basically followed a protocol described before [31], with slight modification. We first collected ca. 10 million cells for each clone and fixed them in 2% paraformaldehyde for 10 min. Then, the cells were lysed in lysis buffer (50 mM Tris (pH 7.5), 150 mM NaCl, 5 mM EDTA, 0.5% NP-40, 1% Triton X-100, 1 × complete proteinase inhibitors (Roche, Cat. 11697498001); 1 ml), passed through a 23-gauge needle, collected and frozen in liquid nitrogen. After the cells were resuspended and treated with 0.3% SDS and 2.5% Triton X100 at 37 °C for 1 h, respectively, we performed first digestion of the chromatin with 200 units of *Nla*III restriction enzyme (New England Biolabs, Cat. R0125) at 37 °C for overnight. After heat inactivation of *Nla*III, the digested chromatin was self-ligated in the presence of 50–100 units of T4 DNA ligase (Thermo Fisher Scientific, Cat. EL0014). After de-cross-linking and purification, we carried out second digestion with 50 units of *Dpn*II restriction enzyme (New England Biolabs, Cat. R0543). Then, the chromatin was again self-ligated with 50–100 units of T4 DNA ligase. After purification with NucleoSpin Gel and PCR purification kit (Macherey-Nagel, Cat. 740609), we measured the concentration of the library using QUBIT 2.0 (Thermo Fisher Scientific). Typically, we obtained 30–40 μg of DNA library from one preparation. We then performed two steps of PCR amplification. The primers used for the first and second PCR are listed in Additional file 2: Tables S6 and S7. The first PCR was to capture and amplify fragments contacting with a viewpoint, with primers targeting there. The amount of template DNA was ca. 3 μg, and the number of cycles was 24. After purification of the PCR product, we carried out eight cycles of the second PCR in order to add adaptor sequences for high-throughput sequencing on the Illumina platform, together with index sequences at the both ends for de-multiplexing of multiplexed libraries. The sequencing platform was HiSeq 2500 (Illumina) for most of the library and MiSeq (Illumina) for the rest (see Additional file 2: Table S7). Though the sequencing was carried out with the paired-end protocol, we used only sequences read from the second cutter for the subsequent analysis. All of the libraries, except the ones from VP-Bmp7 and VP-TZmid2, were prepared in replicates that were processed independently from the collection of the cells.

### Data analysis of 4C-seq

We first combined the separately produced fastq files of same libraries from different lanes. Then, we removed the sequences of the target fragment up to the restriction site with FASTX-Toolkit and mapped the remaining sequences against the mouse genome (mm9) using Bowtie2 software with its default setting [63]. We converted the generated SAM files to BAM files, and then indexed and sorted them using SAMtools [64]. In order to visualize the mapped reads as genome tracks, we normalized the counts as reads per million (RPM), smoothed them with the window size of 11 fragments and finally produced BedGraph files with FourCSeq [65]. We counted reads mapped to given intervals with BEDTools (version 2.26.0) [57]. For the counting, we excluded from the analysis reads mapped within 10-kb (15-kb distance only for the Tdom viewpoint to avoid a large unmappable region nearby) distance from the viewpoints. The coordinates of genome intervals for the analysis are listed in Additional file 2: Table S8. We used Integrated Genomics Viewer [66] to represent the BedGraph tracks and the ggplot2 package (http://ggplot2.org) for R (https://www.r-project.org) to produce plots based on the counts of the 4C-seq reads. The one-sided permutation test was performed with the coin package (https://cran.r-project.org/web/packages/coin/index.html) for R to test the significance of the increase in inter-domain contacts in the mutation alleles from the wild-type allele. One-way ANOVA with Tukey’s multiple-comparison post hoc test was carried out with programs included in R as default. The numbers of replicates were two or three for each group in these statistical tests.

## Additional files

**Additional file 1. Figure S1.** 4C-seq plots of Hap, del1, del2 and del3. **Figure S2.** 4C-seq plots of mutant alleles around the TZ from the VP-Fam209 and VP-Spo11. **Figure S3.** Inter-species comparison of contact domains, CTCF binding pattern and non-coding sequences. **Figure S4.** CRISPR target IDs used to produce the deletion and inversion alleles analyzed in the study.

**Additional file 2. Table S1.** List of ENCODE files of the CTCF binding peaks used in the study. **Table S2.** List of the CRISPR target sequences and their genomic coordinates, using the mm9 assembly. **Table S3.** Combination of CRISPR targets used to introduce mutations in this study. The IDs of the targets are given in Additional File 2: Table S2. See also Additional File S1: Figure S4. **Table S4.** Primer pairs used to confirm the CRISPR mutations. **Table S5**. Primer pairs used for the qPCR assay. **Table S6.** Primers used to prepare the 4C-seq libraries. **Table S7.** List of the 4C-seq libraries prepared and analyzed in this study. The IDs of primers used for the 1st and 2nd PCR are given in Additional File 2: Table S6. **Table S8.** List of intervals utilized for counting mapped reads, and their coordinates using the mm9 assembly.

## Abbreviations

CTCF: CCCTC-binding factor
TZ: transition zone
ES cells: embryonic stem cells
4C-seq: circular chromatin conformation capture assay followed by high-throughput sequencing
VP: viewpoint of 4C-seq
N-ChIP: native chromatin immunoprecipitation followed by qPCR assays
