# Supplementary material for: Control of directionality of chromatin folding for the inter- and intra-domain contacts at the Tfap2c–Bmp7 locus
Source: Epigenetics Chromatin. 2018 Sep 14;11:51. doi: 10.1186/s13072-018-0221-1 (PMC6137755; doi:10.1186/s13072-018-0221-1)
Supplement: Supplementary file 1 — Additional file 1. Figure S1. 4C-seq plots of Hap, del1, del2 and del3. Figure S2. 4C-seq plots of mutant alleles around the TZ from the VP-Fam209 and VP-Spo11. Figure S3. Inter-species comparison of contact domains, CTCF binding pattern and non-coding sequences. Figure S4. CRISPR target IDs used to produce the deletion and inversion alleles analyzed in the study. [file 13072_2018_221_MOESM1_ESM.pdf]

Additional File 1

Control of directionality of chromatin folding for the inter- and intra-domain contacts at the *Tfap2c-Bmp7* locus.

Taro Tsujimura[1,2]*, Osamu Takase[1,2], Masahiro Yoshikawa[1,2], Etsuko Sano[1,2], Matsuhiko Hayashi[3], Tsuyoshi Takato[4,5], Atsushi Toyoda[6], Hideyuki Okano[2], Keiichi Hishikawa[1,2]*

[1]Department of iPS Cell Research & Epigenetic Medicine, [2]Department of Physiology, and [3]Apheresis and Dialysis Center, Keio University School of Medicine, 35 Shinanomachi, Shinjuku-ku, Tokyo 160-8582, Japan
[4]Division of Tissue Engineering and [5]Department of Oral and Maxillofacial Surgery, University of Tokyo Hospital, Bunkyo-ku Hongo 7-3-1, Tokyo 113-8655, Japan
[6]Center for Information Biology, National Institute of Genetics, 1111 Yata, Mishima, Shizuoka 411-8540, Japan

List of Supplementary Tables (in Additional File 2)

Table S1. List of ENCODE files of the CTCF binding peaks used in the study.

Table S2. List of the CRISPR target sequences and their genomic coordinates, using the mm9 assembly.

Table S3. Combination of CRISPR targets used to introduce mutations in this study. The IDs of the targets are given in Additional File 2: Table S2. See also Additional File S1: Figure S4.

Table S4. Primer pairs used to confirm the CRISPR mutations.

Table S5. Primer pairs used for the qPCR assay.

Table S6. Primers used to prepare the 4C-seq libraries.

Table S7. List of the 4C-seq libraries prepared and analyzed in this study. The IDs of primers used for the 1st and 2nd PCR are given in Additional File 2: Table S6.

Table S8. List of intervals utilized for counting mapped reads, and their coordinates using the mm9 assembly.

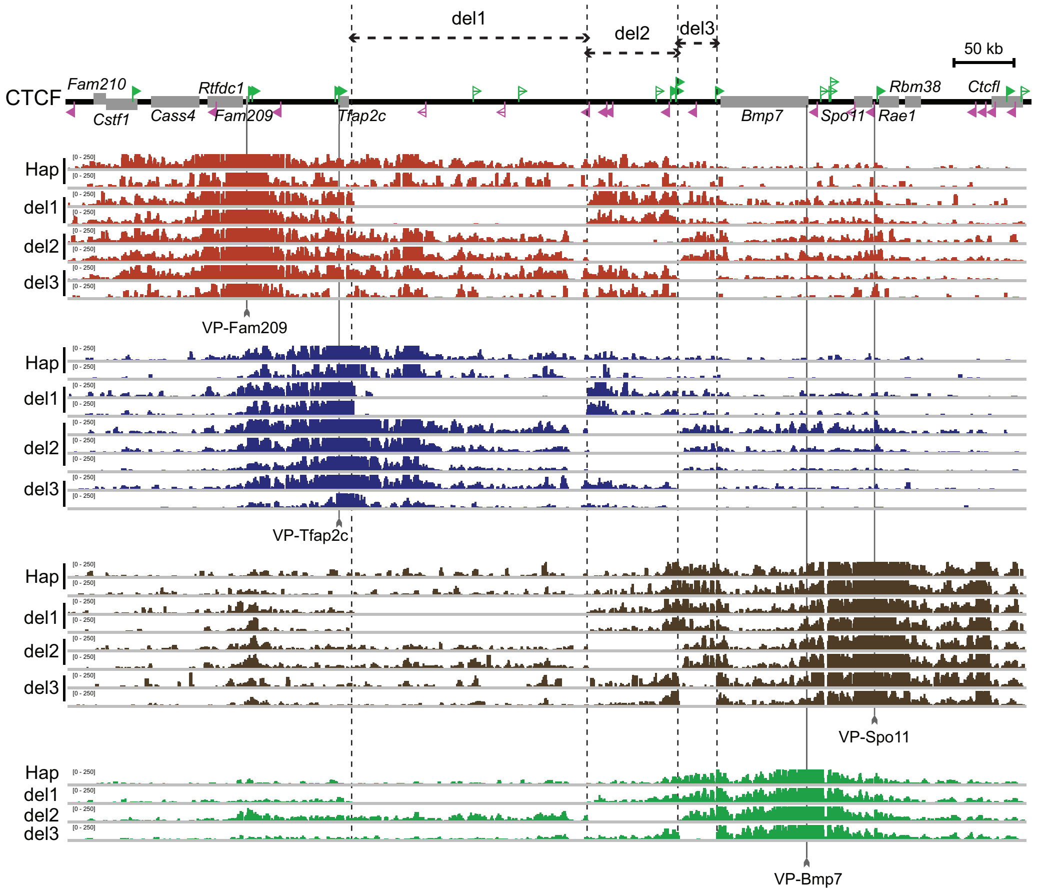

Figure S1. 4C-seq plots of Hap, del1, del2 and del3.

All the replicate results of the 4C-seq plots from the VP-Fam209 , VP-Tfap2c VP-Spo11 and VP-Bmp7 of the Hap, del1, del2 and del3 alleles.

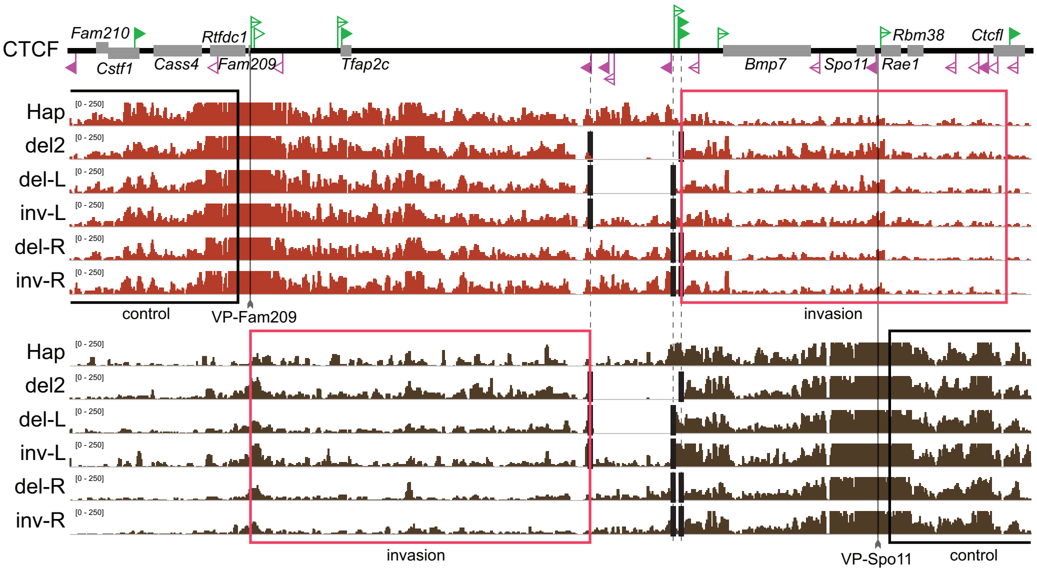

Figure S2. 4C-seq plots of mutant alleles around the TZ from the VP-Fam209 and VP-Spo11.

The 4C-seq profiles from the VP-Fam209 and VP-Spo11 in the different mutant clones: Hap (wild type), del2, del-L, inv-L, del-R, and inv-R. The rectangles indicate areas where mapped reads were counted to score the inter-domain contacts by normalizing "invasion reads" (red rectangles) over "control reads" (black rectangles) in Figure 4b.

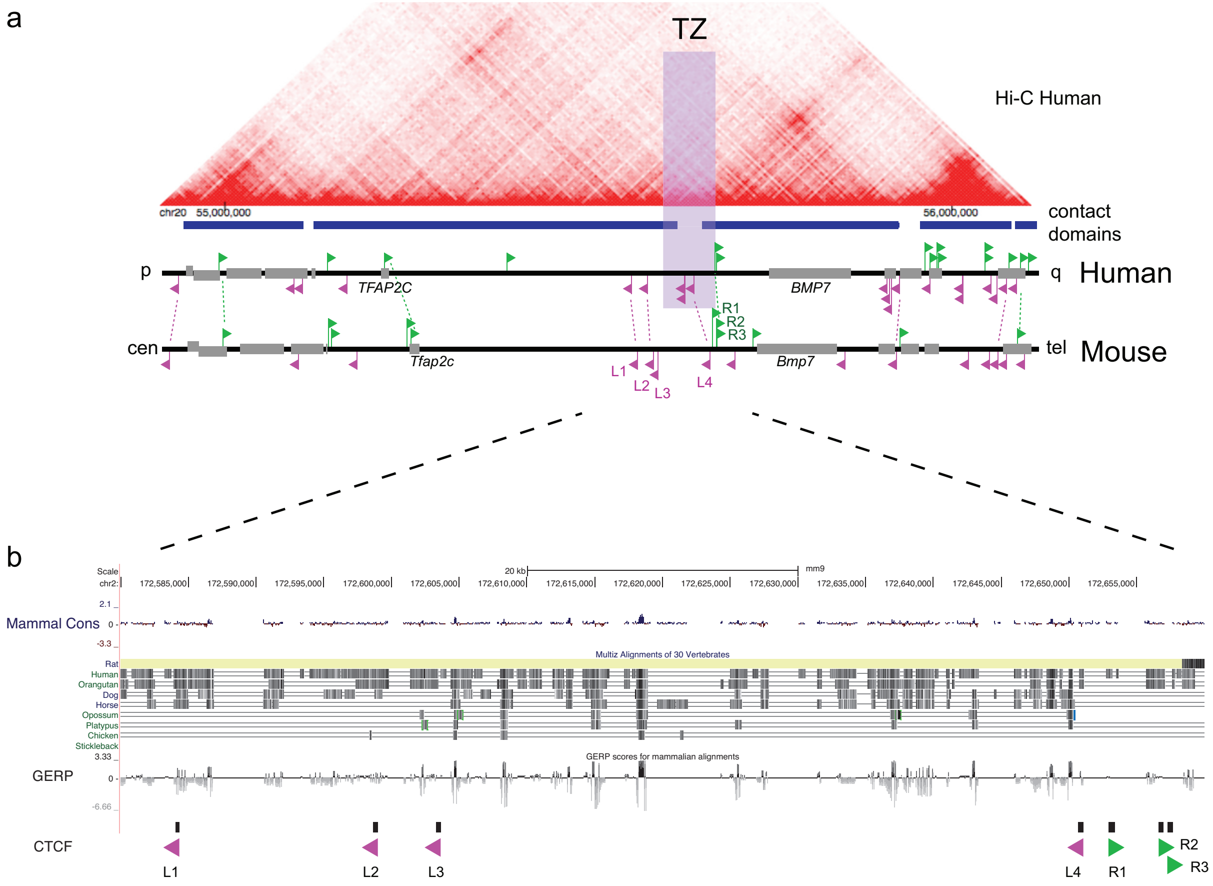

Figure S3. Inter-species comparison of contact domains, CTCF binding pattern and non-coding sequences.

(a) Hi-C domains and CTCF bindings at the *Tfap2c-Bmp7* locus of the human genome (chr20:54910000-56110000, hg19). The left points to the p arm, while the right points to the telomere of the q arm. The Hi-C heat map is based on the data of GM12878 cells at the resolution of 5 kb {Rao:2014eo}. Contact domains called from these Hi-C data are depicted by blue bars {Rao:2014eo}. Note that the *TFAP2C* and *BMP7* domains are consistently called as in the mouse genome with border corresponding to the TZ. The CTCF binding sites are represented as in Figure 1b. The non-tissue-specific CTCF binding sites in the mouse genome are aligned below. The orthology of the CTCF sites between the two genomes is indicated by dashed lines. (b) The UCSC Genome Browser tracks showing the sequence conservation within the TZ (chr2:172580000-172660000, mm9). Note there are several regions conserved in the amniotes or mammals between the TZ-L3 and TZ-L4.

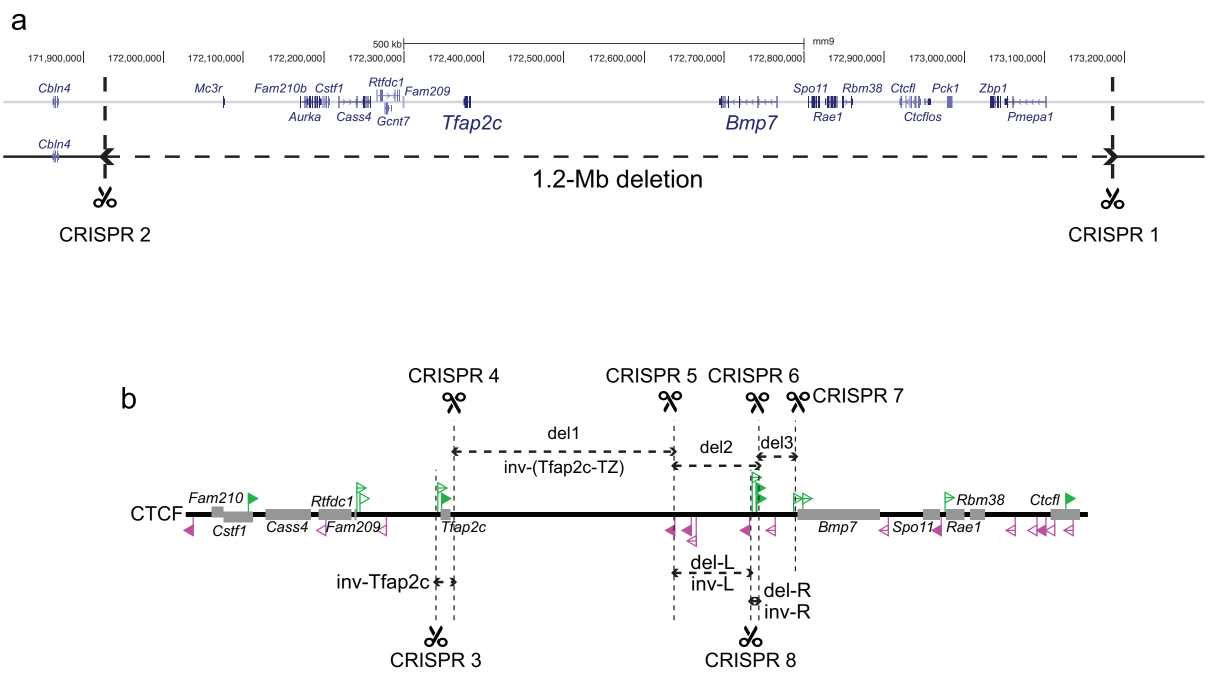

Figure S4. CRISPR target IDs used to produce the deletion and inversion alleles analyzed in the study.

(a) CRISPR targets used to produce the Hap clone with the 1.2-Mb deletion. (b) CRISPR targets used to produce the deletion and mutation alleles. Note that always a pair of two targets were transfected to produce each allele. The numbers correspond to the IDs of CRISPR targets listed in Additional File 2: Tables S2 and S3.
